# Correctness and response time distributions in the MemTrax continuous recognition task: Analysis of strategies and a reverse-exponential model

**DOI:** 10.3389/fnagi.2022.1005298

**Published:** 2022-11-03

**Authors:** J. Wesson Ashford, James O. Clifford, Sulekha Anand, Michael F. Bergeron, Curtis B. Ashford, Peter J. Bayley

**Affiliations:** ^1^War Related Illness and Injury Study Center, VA Palo Alto Health Care System, Palo Alto, CA, United States; ^2^Department of Psychiatry and Behavioral Science, Stanford University, Palo Alto, CA, United States; ^3^Department of Psychology, College of San Mateo, San Mateo, CA, United States; ^4^Department of Biological Sciences, San José State University, San Jose, CA, United States; ^5^Department of Health Sciences, University of Hartford, West Hartford, CT, United States; ^6^MemTrax, LLC, Redwood City, CA, United States

**Keywords:** Alzheimer’s disease, cognition, cognitive impairment, dementia, episodic memory, memory, response time, recognition

## Abstract

A critical issue in addressing medical conditions is measurement. Memory measurement is difficult, especially episodic memory, which is disrupted by many conditions. On-line computer testing can precisely measure and assess several memory functions. This study analyzed memory performances from a large group of anonymous, on-line participants using a continuous recognition task (CRT) implemented at https://memtrax.com. These analyses estimated ranges of acceptable performance and average response time (RT). For 344,165 presumed unique individuals completing the CRT a total of 602,272 times, data were stored on a server, including each correct response (HIT), Correct Rejection, and RT to the thousandth of a second. Responses were analyzed, distributions and relationships of these parameters were ascertained, and mean RTs were determined for each participant across the population. From 322,996 valid first tests, analysis of correctness showed that 63% of these tests achieved at least 45 correct (90%), 92% scored at or above 40 correct (80%), and 3% scored 35 correct (70%) or less. The distribution of RTs was skewed with 1% faster than 0.62 s, a median at 0.890 s, and 1% slower than 1.57 s. The RT distribution was best explained by a novel model, the reverse-exponential (RevEx) function. Increased RT speed was most closely associated with increased HIT accuracy. The MemTrax on-line memory test readily provides valid and reliable metrics for assessing individual episodic memory function that could have practical clinical utility for precise assessment of memory dysfunction in many conditions, including improvement or deterioration over time.

## Introduction

### Need for screening tools for cognitive and memory impairment

Cognitive impairment often includes dysfunction of episodic memory and is currently recognized as one of the most widespread and challenging public health problems of our times. Episodic memory impairment is a hallmark of Alzheimer’s disease (AD), and the rates of dementia and AD are rapidly increasing with aging populations. Traumatic brain injuries, drug side-effects, anesthesia, and many other untoward events often disrupt brain function with consequent cognitive and memory dysfunction. Accordingly, to develop early detection and monitoring change over time as key strategies in managing this escalating burden on society, assessment of cognitive function, particularly episodic memory, is critically important.

Currently, there is a significant and recognized lack of adequate instruments for brief cognitive screening and early detection of the dementia caused by AD (Ashford, 2008; De Roeck et al., 2019; Ashford et al., 2022). Most tools for assessing cognitive function require face-to-face administration using paper-and-pencil instruments, which are complex, labor-intensive, and subject to intra-rater and inter-rater variability. In the current era, computerized assessments for measuring cognition, including episodic memory, are a viable alternative, though, at this time, no test has distinguished itself as generally useful (Zygouris and Tsolaki, 2015; Sternin et al., 2019). With computerized testing, several aspects of cognitive performance can be quantified much more precisely, including correctness (accuracy) of performance and response time (RT). This study focused on a continuous recognition task (CRT), which can be efficiently implemented for memory screening for a broad range of cognitive problems including AD.

The CRT should be clearly distinguished from a similar task paradigm, the “n-back” test, which traditionally uses a limited number of stimuli (e.g., numbers or letters) and asks the subject to recall if a stimulus is a repeat from a stimulus 2, 3, or 4 back. This latter test (n-back) is useful for examining working memory (WM) and executive function, and there are several n-back tests in use, including one that is computerized (Pelegrina et al., 2015). However, in the CRT examined here, few repeated images occurred less than four images later and thus require an absolute recognition for detection, given there were five categories and five images from each category, exceeding the brains usual capacity to rely on WM. Accordingly, the CRT is substantially different than the usual n-back test.

### Continuous recognition task history and signal detection theory

The present study analyzed the distribution of behavioral data produced when individuals performed an online modification of a traditional Signal Detection Task (SDT), the MemTrax CRT (Ashford et al., 2019b), to test learning, memory, and cognition. Prior in-person, not-computerized CRTs, the Continuous Visual Memory Test (Larrabee et al., 1992) and the Continuous Recognition Memory test (Fuchs et al., 1999), have used a similar format with different types of stimuli and have shown that the CRT format has construct validity. Related studies have examined the retrieval processes involved in continuous recognition (Hockley, 1982) and provided information on memory trace strength and the memory theories (Hockley, 1984b,^2022^). Further, similar continuous recognition tests have shown the involvement of the frontal, parietal, and temporal cortical regions and the hippocampus in the performance of this type of memory task (Yonelinas et al., 2005; Suzuki et al., 2011), brain regions affected in AD and most types of dementia and cognitive and memory impairment. Accordingly, the MemTrax CRT was expected to provide valid data for several aspects of memory, learning, and cognition, though many other aspects, such as free recall, verbal, semantic, and remote memory would not be assessed.

Whereas most SDTs direct participants to attend to and detect a designated “target” stimulus (single or previously defined item or items), the MemTrax CRT instructs participants to consider all presented stimuli and detect a repetition of any stimulus in the randomized sequence and indicate that detection with a response (a space-bar press, a screen tap, or a mouse click referred to commonly as a “HIT”). Failure to recognize a repeated stimulus is a “Miss.” The consideration of an initial stimulus being shown without responding, for 3 s to “learn” the new information, is referred to as a “Correct Rejection,” while responding to the first presentation of an image in this case would be considered a “false alarm.” The primary hypothesis in this study was that SDT analytic methodology (Stanislaw and Todorov, 1999), distinguishing repeated images (signals) from initial presentations (non-signals), applied to the MemTrax output data would provide two specific metrics, the degree of correctness of performance (percent correct, reflecting the *d’* – *d*-prime - component) and the tendency to over or under respond (response balance or bias of HITs and Correct Rejections, reflecting the “beta” component), and these metrics were anticipated to differentiate cognitive function and thus explain individual performance on this CRT. The secondary hypothesis was that RT would correlate most directly with correctness of performance (related to d’), rather than the response balance, a speed-accuracy trade-off (related to beta), or percent HITs or Correct Rejections. In a prior version of MemTrax, using a PowerPoint presentation to audiences, *d’* correlated with age (*r* = –0.37), more than HIT rate (*r* = –0.24) or false alarm rate (*r*^2^ = –0.25), though RT was not available (Ashford et al., 2011). However, in another study of MemTrax on-line, the correlation of percent correct with age was much less (*R*^2^ < 0.02), while the correlation of RT with age was about (*R*^2^ = 0.08) (Ashford et al., 2019b); percent HITs and Correct Rejections were not analyzed in that study.

A predecessor of the MemTrax CRT was used in a primate laboratory (Ashford and Fuster, 1985; Coburn et al., 1990), where visual cortical neuron response latencies to information-laden stimuli were found to occur simultaneously across recruited cortical regions, suggesting a coordinated massive reciprocal capacity for item analysis (Ashford et al., 1998a). It was further shown that the Rhesus monkeys could recognize letters in a serial visual learning and recognition task (Ashford et al., 1998a). This serial visual recognition task was later modified for clinical use with a slide projector using complex visual stimuli and then piloted as a PowerPoint presentation to large community-based audiences of elderly individuals concerned about their memory (Ashford et al., 2011). Because of the engaging nature and positive user experience reported, this task paradigm was implemented online to assess memory problems in the general population (Ashford et al., 2019b). This sophisticated but simple paradigm can be quickly administered with measurement precision far beyond that possible with paper-and-pencil tests (van der Hoek et al., 2019; Liu et al., 2021).

### Episodic memory and response time to recognize visual stimuli

Episodic memory contains the ‘what,’ ‘where,’ and ‘when’ information that interacts and binds with information in semantic memory to form time-based concepts of those events. The organization of these defining declarative features into progressively more complex concepts optimizes capacity limitations imposed on short-term memory (STM). Recall of information from non-declarative, or implicit memory requires no conscious or intentional involvement and is referred to as perceptual memory. Thus, in contrast to content-based storage in the episodic, semantic, and declarative systems, implicit memory includes processes and procedures that reduce effort to learn, store, think about, and convert information in STM into long-term memory (LTM). An important concept is the efficiency with which information can be integrated across such processes (Weigard and Sripada, 2021).

Response time to stimuli presented in tasks has been studied extensively in evaluating episodic memory. Recognition memory is an area of notable interest. Whereas paired-associates learning provided an early method to study memory (Shepard, 1958), recognition testing provides a method for estimating the quantity of information retained in memory (Shepard, 1961). Recognition memory paradigms have been studied to determine memory capacity and limitations, particularly using SDT and speed-accuracy trade-offs, and comparing memory theories such as the recruitment model and scanning model, with important implications for decision latency, including correct responses which are shorter than incorrect responses (Pike et al., 1977). An important advance was the development of a continuous recognition approach in which new and old items were interspersed (Shepard and Teghtsoonian, 1961). Since the early studies of recognition performance, demonstrations of the utility of complex pictures in the CRT paradigm to study memory have been extensive.

Surprisingly, the first scientific study conclusively demonstrating that the human could remember large amounts of information utilized numerous complex color pictures presented to individuals who showed high levels of recognition after both short and very long delays (Shepard, 1967). In a cross-species study, pigeons and monkeys are able to recall complex pictures moderately well; however, humans remember pictures so well that to test the limits of normal human capacity, it is necessary to utilize highly complex stimuli, such as kaleidoscope images (Wright et al., 1985). With such recognition memory paradigms, RT to stimuli can be analyzed to determine the time which the individual takes to recognize and respond to a previously shown item (Hockley, 1982).

A prominent interest has been the decay of memory traces coincident with increasing intensity of intervening distractions (Hintzman, 2016), including the lag from a first to a second presentation of an image (Hockley, 1982). Complex picture recognition has been particularly useful for studying medial temporal lobe function (Suzuki et al., 2011; Koen et al., 2017). And recognition memory and CRT paradigms have been used effectively in studying neural responses in the human hippocampus to assess episodic memory (Wixted et al., 2018), regardless of variations in the method of test administration (Bayley et al., 2008). However, behavioral pattern separation in memory progressively and distinctively declines from healthy individuals to those with mild cognitive impairment (Stark et al., 2013).

The MemTrax CRT requires complex picture information processing into STM and access and recognition of content from LTM for use in responding to the future stimuli presented in the task, because each stimulus, in addition to being a potential current target, is also potentially a new stimulus and thus a potential later target. CRTs like MemTrax are applied to examine these events in the brains of subjects instructed to attend to stimuli and indicate repetition. In this case, detection of repetition of the “target” stimulus produces an overt behavior (response, either a space-bar press, a screen touch, or a mouse click) that signals “yes, a repetition was detected” or a covert behavior (no response) indicating “no, a repetition was not detected” on a particular trial.

### Response accuracy, time, and factors modifying signal detection

Signal detection task suggests that there are two factors which affect the accuracy of information processing and the time to accurately respond to a stimulus (RT) as instructed. The first factor is the internal state of the subject related to their health and prior knowledge stored in LTM. This factor relates to the motivation to participate in the testing, the ability to sense the stimulus and maintain the instructional set, and issues not related to the task. Internal state in this context alters the ability of the subject’s information processing sequence to engage with the task and execute a correct behavior (HIT: target present and participant responds as instructed; and Correct Rejection: target not present and participant does not respond) or an incorrect behavior (False Alarm: target not present and participant responds in spite of instruction not to respond; and Miss: target is present, and participant does not respond as instructed) on each trail. This ability factor is referred to as *d’* (*d*-prime) and reflects the sensitivity or degree of discrimination between the targets and non-targets. Accordingly, in a CRT, the SDT models a single factor controlling recognition correctness but does not account for different internal or external issues which may differentially affect the HIT versus Miss recognition or the Correct Rejection versus False Alarm decision.

The second factor is the knowledge acquired by the subject performing the task about environmental factors, like the *a priori* probability of a particular occurrence and the payoff matrix describing the consequence for a correct versus incorrect behavior on a trial (Liu et al., 2019). Such knowledge can be used by cognitive processes directed by those operations in WM to establish a criterion for response performance on subsequent trials during the task. This second factor is referred to as “beta,” reflecting the tendency to under-respond or over-respond. The predilection to miss targets (recognition failure) or wrongly identify new stimuli (incorrectly guess, False Alarm) is of great importance for interpreting performance and understanding disorders of a subject’s information processing system and is related to the RT (Gordon and Carson, 1990). However, there may be many factors involved in the processing of information and cognitive impairment. So, the process of recognition may be impaired and slow responsiveness, while a separate, unrelated executive process may change the response bias and affect RT in a different manner.

Instructions provided to the participant prior to testing describe the processing required to meet task demands for a CRT (Craik, 2002). These instructions direct the required operations in WM on how to execute processes to meet task demands. In the MemTrax CRT, the neurocognitive processes are: (1) specifically compare and detect representation(s) that match prior occurrences during the test, e.g., “recognize”; (2) if a recognition occurs, manifest a response as quickly as possible; and (3) direct processes to use information on a trial to update expectations so this information is adequately encoded to be available for “recognition” for subsequent trials (Walley and Weiden, 1973; Fabiani et al., 1986; Clifford and Williston, 1992, 1993). As described for attention (Posner, 1994), instructions may alter the effects which the internal state of the subject has on processing during and between trials. Complex visual information, as shown to participants during a MemTrax trial, activates visual cortical regions, including the occipital and inferotemporal cortex (Ashford and Fuster, 1985). However, the MemTrax test instructions require attention to the stimuli for recognition and possible encoding, which will also activate the prefrontal cortex (Kapur et al., 1994; Ashford et al., 1998a).

### Response time distribution skewing

The present study examined RT means and their relationship to the universal observation that the averaged distribution of RTs during CRTs differs from the normal Gaussian distribution and is skewed, with absolute lower limits and less bounded upper limits. The explanation for the skewed distribution of RTs has been difficult, though numerous models have been suggested (Burbeck and Luce, 1982; Hockley, 1982, 1984a; Moret-Tatay et al., 2018, 2021; Liu et al., 2019). An exponentially modified Gaussian probability density function (ex-Gaussian) (Ratcliff and Murdock, 1976), which provides parameters related to performance across different tests (Hockley, 1984a), has been widely used to model individual RTs. The ex-Gaussian model is based on a theory that RT reflects two underlying psychological mechanisms (processes): the decision component for sensory processes that obeys an exponential distribution (decay curve), and the transduction component related to the initiation and completion of the physical response to the stimuli that follows a normal Gaussian distribution (Dawson, 1988; Marmolejo-Ramos et al., 2014). Following this theoretical construct, RT during these tasks has been modeled as the convolution of an exponential function and a Gaussian function to form an exponentially modified Gaussian curve (the ex-Gaussian function) sensitive to both mechanisms. The ex-Gaussian function has been invoked to explain recognition processing based on RTs (Moret-Tatay et al., 2021) and reaction time slowing in AD (Gordon and Carson, 1990; Ratcliff et al., 2021). This distribution requires three parameters to model RT, the mean and standard deviation of the Gaussian distribution and the decay constant of the exponential component.

Due to issues related to the complexity of RT measurement and explanations of the underlying neural mechanisms subserving decision making, significant effort has been expended to develop other approaches to model the skewed distribution of RTs to obtain a deeper understanding of experimental effects on the underlying neural and psychological processes supporting these data (Ratcliff et al., 2016; Weindel et al., 2021). These approaches have been divided into two groups, measurement models and process models (Anders et al., 2016; Tejo et al., 2019). The measurement models include Weibull and lognormal (Anders et al., 2016) models. The process models, which address the internal information analysis by the individual, include the diffusion model (Ratcliff and Murdock, 1976; Ratcliff and McKoon, 2008; Ratcliff et al., 2016; Liu et al., 2022) and the leaky-competing accumulator model (Usher and McClelland, 2001; Tsetsos et al., 2012). In reality, there are many mathematical models that can produce similar distributions to explain various complexities of cognitive tasks (Liu et al., 2019), and many theoretical models have attempted to provide explanations (Cousineau et al., 2016; Osmon et al., 2018; Hasshim et al., 2019), though the actual neural processing may not conform to such models.

### MemTrax continuous recognition task analysis

In the present study the distributions of correct and incorrect behaviors were examined with respect to overall performance. Then, the distribution of mean RTs executed during the MemTrax CRT was evaluated. The objective of the present analyses was to determine the extent to which the cumulative distribution of performance metrics during this test, number of responses, correctness of responses, and the RT for correct responses, can be modeled, with determinations of types of performance limitations and interactions. Of particular interest were response performance levels, response biases (strategies, value-based decision-making), and the relationship of RTs to an exponential regression, which only requires two parameters. There was an earlier expectation that there would be a speed-accuracy trade-off, but prior studies showed that correctness of performance has a slight positive correlation with RT in various populations. Also, previous research examined percent correct and presumed that there would be a close relationship between HITs and False Alarms. Further, it was expected that both types of behavioral responses and RTs would be balanced, reflecting the central processing of information, consistent with an information processing model (IPMs) using SDT. While these expectations were not correct, these analyses in a large group of subjects provided new behavior models and solid bases for using the MemTrax CRT for more extensive assessments and reliable interpretation of behavioral performance. The data, though from anonymous individuals, clearly showed a distribution of several behavioral metrics and provided a guide to determine normal ranges. Metrics from such a large population can lead to the establishment of valid and reliable assessments of episodic memory function in clinical settings.

## Materials and methods

### Population

**FIGURE 1:**
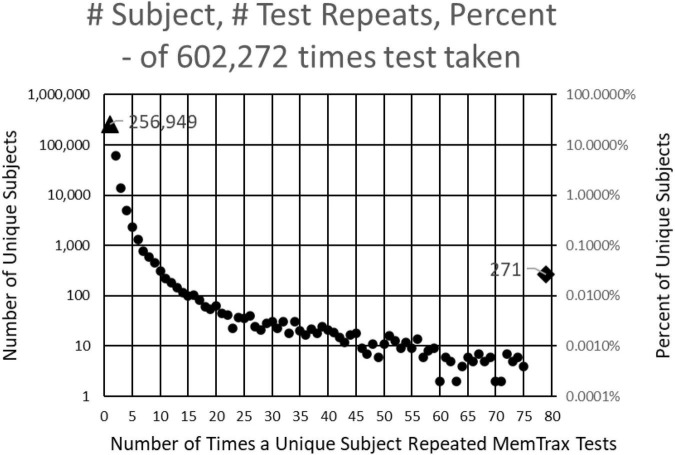
Number of subjects **(left log scale)** and percentage **(right log scale)** taking each number of tests. 344,165 presumably unique individuals took the test. Of these, 256,949 took the test only one time, and 60,642 took the test only two times. Two hundred and seventy-one took the test more than 75 times and 18 took the test more than 1,000 times.

This study examined results from individuals who completed the MemTrax automated CRT program: https://memtrax.com on the internet between May 27, 2014, and May 7, 2022. During this time, over 2 million hits were recorded on the MemTrax website. Of these, the test was started and completed 602,272 times by 344,165 distinct users. The test was programmed to save data on the server before the test results were returned to the user. First-time users were offered an option to sign-up on the website and have their data associated with their password protected email account so that they may see their own performance over time. Of these users, 256,949 took the test only once, while 87,214 (25%) signed up for and took repeat tests. Of those who signed up, 271 of these users took the test more than 75 times and 18 took it more than 1,000 times (Figure 1). For this analysis, data were examined for only the first test for first-time users who took and completed the test, presumably 344,165 unique individuals.

At sign-up, subjects were asked to provide year and month of birth, sex, and education level, though there was no method for verification of this information. This demographic information was only provided by 26,834 users; however, not being verifiable, these data were discarded. Data from all tests, including RT for each individual stimulus (50), were available for analysis, though only average RT was examined in this study. The Stanford University Internal Review Board approved this test for anonymous collection and analysis of these data.

### Design

The MemTrax test program was designed in WORD-PRESS so that it would perform essentially the same on any platform. The on-line implementation presents 50 images, 25 new and 25 repeated, with the instruction to respond to repeated images as quickly as possible. Users are allowed up to 3 s after stimulus presentation to respond to an image. The exact time for each response (1–3,000 ms) was recorded, with 200–2,999 ms considered a “response” and less than 200 ms or exactly 3,000 ms considered a “non-response.” (The 200 ms lower limit was chosen as a typical RT for a fast-reaction to a stimulus change – a value well below any observed decision time, hence not a legitimate response.) Each test had five unique images from each of five categories, which were selected from 3,000 images curated into 60 categories. These images were presented in a pseudo-random order with no more than four new images or repeated images occurring in a sequence (similar to the rules of the Gellerman series, Gellerman, 1993). For the five items in each of the five categories (25 unique images), three images were repeated once, while one was repeated twice, and one was not repeated. This study did not analyze the effects of lag or number of repeats.

Basic analyses for every individual examined “Responses” either indicated by the press of a space bar, the tap of a screen, or click of a mouse, depending on platform used, for each stimulus and mean RT across all HITs. The number of “Correct Trials” was tabulated (0–50; ideally 25 HITs and 25 Correct Rejections). From these measures, other metrics were calculated, including: number of incorrect responses (“False Alarms” = “25 – Correct Rejections,” optimally zero), number of failures to respond to a repeated image (“Misses” = “25 – Hits,” optimally zero), “Total Number of Responses” (“HITs” plus “False Alarms”), and mean RT to “HITs” (“RT”). A more complete description of the task has been previously published (Ashford et al., 2019b).

### Data analysis

For first-time tests for the 344,165 unique users, 59,499 tests with performance of chance or poorer, i.e., less than 30 correct out of 50 possible choices (random likelihood of getting 30 or more correct is less than 1/1000), which included tests with fewer than five HITs or fewer than five Correct Rejections, were removed (17% of the initial uses), leaving 284,644 tests. Also, of these tests, those with average RTs to HITs less than 0.5 s (2,350 had less than 0.5 s with very few of these having more than 30 correct responses, 0.8%) or more than 2 s (*n* = 156) were removed. Another 18 were removed due to a programming error. Thus, 282,140 tests were considered valid and used for this study and analyses (82% of the first-time users).

Data from these tests were analyzed with an EXCEL spreadsheet (Microsoft, Inc., Redmond, Washington, IL, USA). Main functions used included sorting, scatter plots with trend lines and Pearson correlations, COUNTIF, and AVERAGEIF. Analyses were made according to total overt responses (sum of HITs and False Alarms, based on platform, either space-bar presses, screen taps, or mouse clicks, the dependent variables), in response to the picture stimuli (initial or repeated, the independent variables) and total correct trials (sum of HITs and Correct Rejections). Specific analyses of the numbers of types of responses (HITs, Correct Rejections, Misses, and False Alarms) across subjects were performed. The distribution of RTs was analyzed by examining the cumulative distribution and the negative natural log of the cumulative distribution which was tested for its relationship to an exponential regression. RT was analyzed for its relationship with the performance metrics.

## Results

### Number of responses and response correctness

**FIGURE 2:**
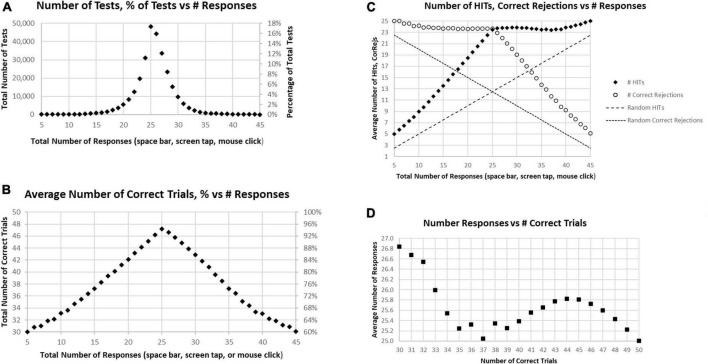
**(A)** Number of tests (left scale) and percentage of tests (right scale) having the specific number of responses. The optimal number is 25, consistent with the peak. **(B)** Average number correct (HITs plus Correct Rejections) of tests (left scale) and percentage of correct responses (right scale). While the number of correct responses ranged from 5 to 45, the optimal number, 25, has only an average number correct equal to 47.2. **(C)** Separated average number of HITs and Correct Rejections plotted for total responses. The pattern is clearly not random (dashed lines); so, when the number of responses is less than 25, the number of HITs declines progressively with a relatively stable number of Correct Rejections, and when the number of responses is more than 25, the number of HITs is relatively stable, with the number of Correct Rejections progressively declining. **(D)** Average number of responses for each number correct, from 30 (60% correct) to 50 (100% correct). Note 25 is optimal, but all averages are above 25 except for 100% correct. Less than 37 correct is associated with an increasing number of responses associated with fewer correct trials.

About 17% of participants had exactly 25 responses (optimal number), with about 32% having less than 25 responses and about 51% having more than 25 responses, which would therefore include correct and incorrect overt responses (Figure 2A). There were approximately equal proportions of correctness for tests with less than 25 total responses and more than 25 responses, with a monotonic decline of correctness with progressively less and more than 25 responses (Figure 2B). This decline was clearly related to a progressive decrease of HITs for tests with less than 25 total responses, with a stable, high number of Correct Rejections. Symmetrically, above 25 total responses, there was a progressive decrease of Correct Rejections, with a stable, high number of HITs, indicating that the proportion of HITs and Correct Rejections was dominantly influenced by a strategy to have either fewer or more responses across all the stimuli, a pattern clearly different from a random interaction (Figure 2C). Though exactly 25 HITs were needed for a perfect score, for subjects with 38–49 correct trials (1–12 errors) there was a slight but clear tendency to respond to more than 25 of the repeated images (more False Alarms than Misses), with the maximum at 44 correct trials, averaging 25.8 responses (Figure 2D). By contrast, subjects executing 35–37 correct trials had active responses to about 25 of the repeated images (an average balance of False Alarms and Misses). Subjects with less than 35 correct trials (more than 15 errors) tended to substantially over-respond (even more False Alarms than Misses) up to an average of 26.6, an over-response rate of 7%. The pattern in Figure 2D indicates a complex relationship between the number of correct trials and responses.

**FIGURE 3:**
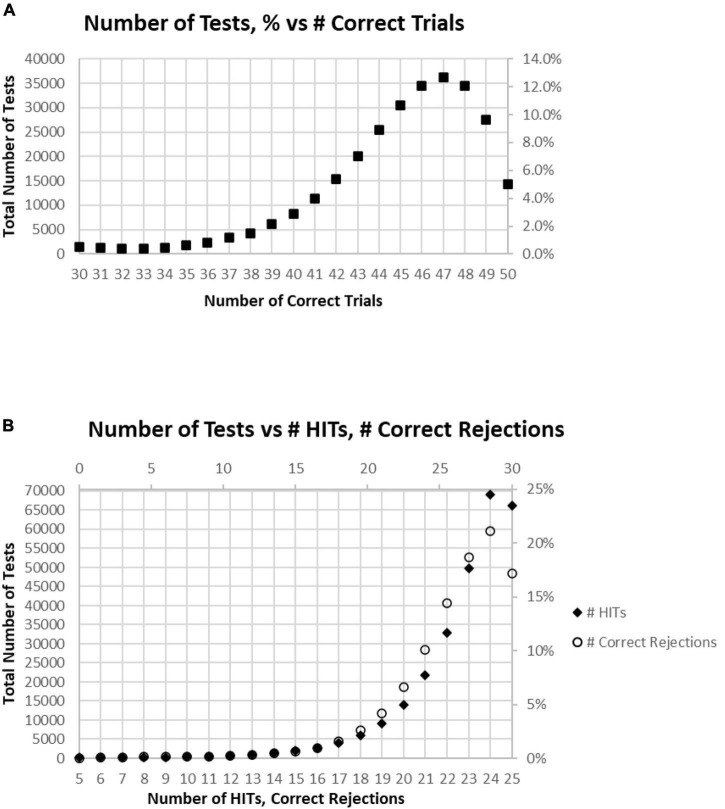
**(A)** Number of tests performed for each number of correct responses. **(B)** Total number of tests broken down to show as HITS and Correct Rejections.

Among these tests, only about 5% of participants had perfect performance (25 correct responses and 25 correct rejections), and 10% of the participants had 49 correct responses and correct rejections (one False Alarm or one Miss; Figure 3A). When the correct components, HITs and Correct Rejections, were plotted separately, they had a similar distribution to the overall correct response plot (Figure 3B). However, the maximum number of HITs for tests occurred at 24, for 24.5% of the tests, while the maximum number of Correct Rejections, also occurred at 24, with 21.1% of the tests. Figure 2C shows the discordance of HITs and Correct Rejections, showing symmetrical variation, but they have a different peak than the correct response total.

**FIGURE 4:**
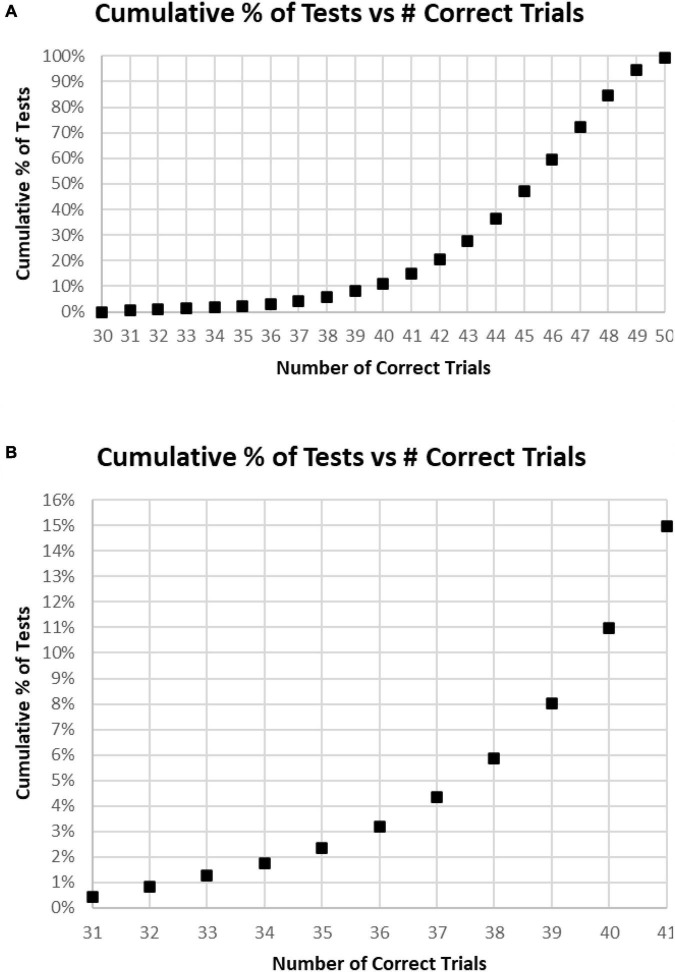
**(A)** Cumulative percentage of tests from 30 correct (60%) to 50 correct (100%). **(B)** Higher resolution of **(A)** to show the percent of tests more precisely for number correct 31–41.

Of all tests, 63% of participants had at least 45 (90%) Correct Responses (HITs and Correct Rejections) on trials with no more than five incorrect responses (False Alarms and/or Misses) (Figure 4A). Of these unique subjects, 85% had at least 42 (84%) correct trials (HITs and Correct Rejections; no more than 10 errors), 92% had at least 40 (80%) correct, while less than 3% had 35 or fewer (70%) correct responses (at least 15 errors) (Figure 4B).

**FIGURE 5:**
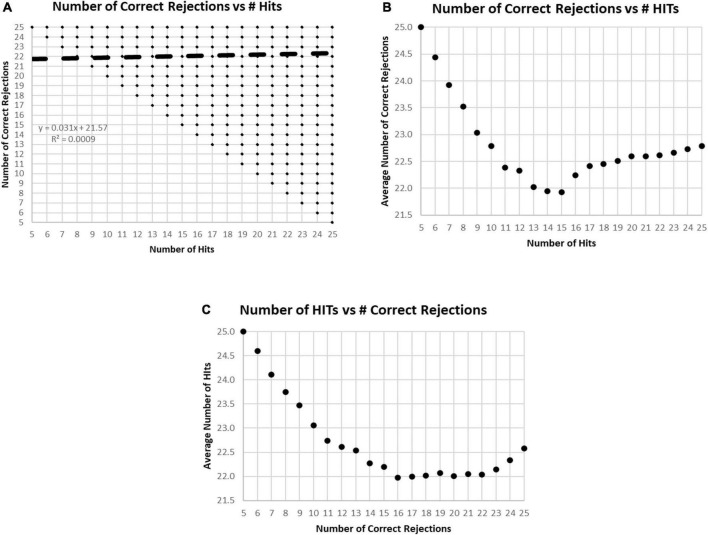
**(A)** Plot of number of Correct Rejections versus number of HITs. The correlation showed essentially no relationship. Among the 282,140 tests, all 231 possible variations of responses were represented. The limitation of five HITs and five Correct Rejections was due to the limitation of the data set to those subjects with at least 60% correct, 30 correct responses. **(B)** Plot of average number of Correct Rejections versus number of HITs. Note the slow decrement of correct rejections with a decrease of HITs from 25 down to 17, at which point, there is a drop of correct rejections, but then below 15 HITs there appears to be a strategy to be more careful have more correct rejections. **(C)** Plot of average number of HITs versus number of Correct Rejections. Note the decrement of HITs from 25 to 22 Correct Rejection, then below 16 Correct Rejections, there is a sharp tendency to have more HITs, which appears to be a strategy to respond more, indiscriminately.

In the examination of the relationship between HITs and Correct Rejections, there was essentially no correlation (*R*-squared less than 0.001) (Figure 5A). When plotting the average number of Correct Rejections versus the number of HITs (Figure 5B), or the average number of HITs versus the Correct Rejections (Figure 5C), the performance above or below 25 responses noted in Figure 2C is clearly explained. The implication of these analyses is that subject strategies have a complex relationship with the manifest performance, including the tendency to respond to more or less than 25 images, which reflects a consistent bias across responses to new and repeated images. Accordingly, the responses of these participants are far from random, and the patterns of their responses presumably represent specific intents, biases, predispositions, or strategies.

### Response time distribution

**FIGURE 6:**
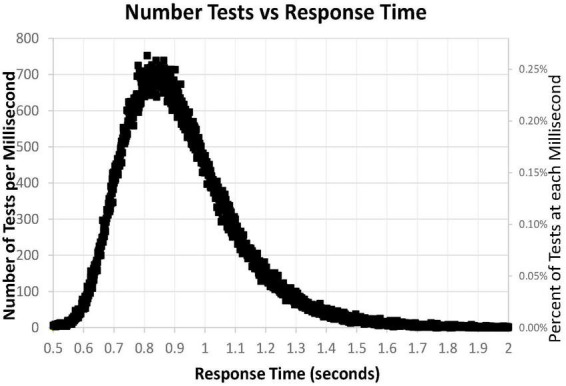
Number of tests for each millisecond RT, from 0.500 to 2.00 s, for 282,140 user tests. Note that the number of tests has a skewed distribution.

**FIGURE 7:**
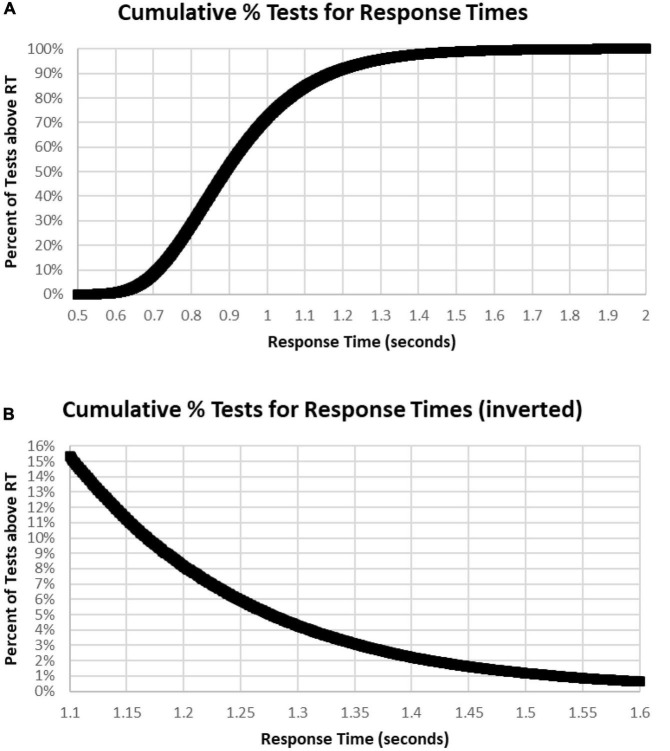
**(A)** Cumulative percentage of tests with respect to RT. Note that level is below 1% until over 0.620 s and 99% had RTs less than 1.570 s. Median RT was 0.900 s, at 50%. **(B)** Inverse of **(A)** at higher resolution to show the slower 15%, more than 1.1 s, and the slower 1%, more than 1.6 s.

A major issue was the distribution of mean RTs for HITs during the MemTrax CRT paradigm. Only mean RTs for HITs between 0.5 and 2 s were considered for this analysis. The distribution of those RTs shows a clear inverted-U-shaped pattern skewed to the right, and the median RT was 0.89 s (Figure 6). Only 63 subjects had RTs for HITs between 0.500 and 0.510 s, six individuals at each millisecond interval, while more than 600 subjects had RTs at each millisecond interval between 0.8 and 0.9 s, a total of 68,550 (24%). The RT cumulative distribution (RTCD) also shows the relationship between RTs and number of tests (Figure 7A). For 2 standard deviation limits, 2.2% of the population had RTs faster than 0.647 s while another 2.2% of the population was slower than 1.4 s. Only 1% of the participants had RTs faster than 0.62 s, and the increase of false alarms for subjects responding in this range (see below) suggests that they were sacrificing accuracy for speed. Only 1% of subjects responded slower than 1.57 s (Figure 7B), and these subjects also generally had lower correct response percentages and fewer HITs (see below). The fast responders were the only participants who appeared to manifest a speed-accuracy trade-off.

The sharp rising slope of fast RTs between 0.5 and 0.7 s, the rounded peak between 0.7 and 1 s, and the prolonged tail of slow RTs beyond 1.2 s (Figure 6) showed the skew of the RTCD in this data. Therefore, the basis of this RTCD skew was considered for development of an explanatory mathematical model. Most equations have difficulty accounting for the sharp drop of the fast RTs and the prolongation of the slowed RTs. Consistent with the limited capacity of the activated neurophysiological mechanisms required for efficient engagement of the information processing sequence (Broadbent, 1965), the RTCD must reflect the time needed for information processing to occur in the neural substrates supporting resources in the visual modality. Certain elements relate to STM, and others involve processes in WM, while the slow decline reflects the lack of such resources for processes directed by operations in WM. A variety of mathematical models have been invoked to explain the skewed distribution seen in RTs. However, these models use at least three parameters to describe the skew distribution. For example, the ex-Gaussian distribution uses two Gaussian parameters and an exponential parameter (Dawson, 1988).

**FIGURE 8:**
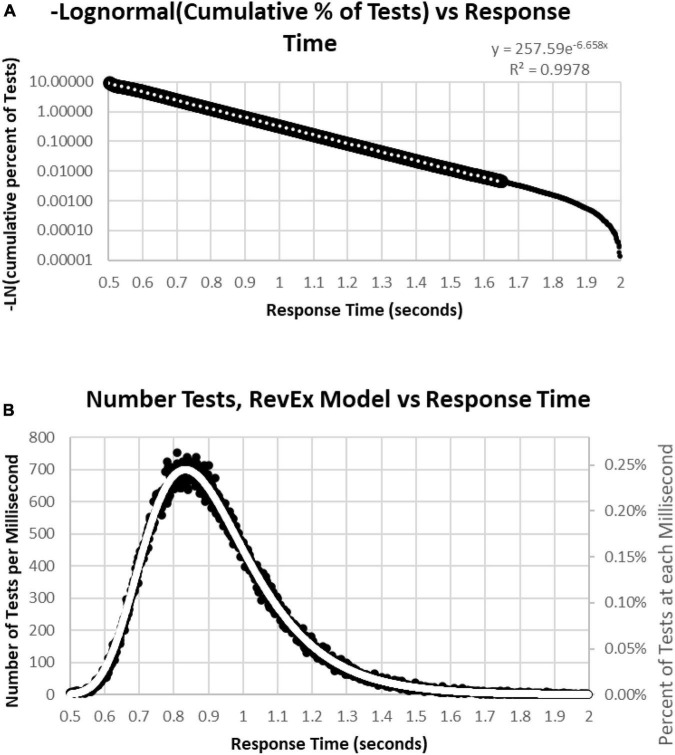
**(A)** Negative natural logarithm of the cumulative percentage of RTs plotted against the RT. The Pearson regression exponential curve explained nearly all the variance between 0.5 and 1.65 s. **(B)** Calculation of exponential curve back to number of RTs. The curve clearly fits the distribution of the RTs. The most responses were at 0.810 s followed by 0.839 and 0.864 s, while the peak of the RevEx model was at 0.833 s.

To better understand the skewed RTCD, a new perspective was taken to describe this RTCD. Examination of the RTCD (Figure 7A) demonstrated a curve similar, but in reverse, to a survival curve, also known as a Gompertz Law exponential hazard function (Hirsch, 1997; Gavrilov and Gavrilova, 2001; Raber et al., 2004). To test the applicability of this mathematical model, a negative normal log of the CD was calculated (Figure 8A). This exponential curve explained nearly all the variance: *R*^2^ = 0.9999:

Ln(CD) = 263.94 × EXP(–6.682 × RT) (Cumulative Distribution; Response Time)

**FIGURE 9:**
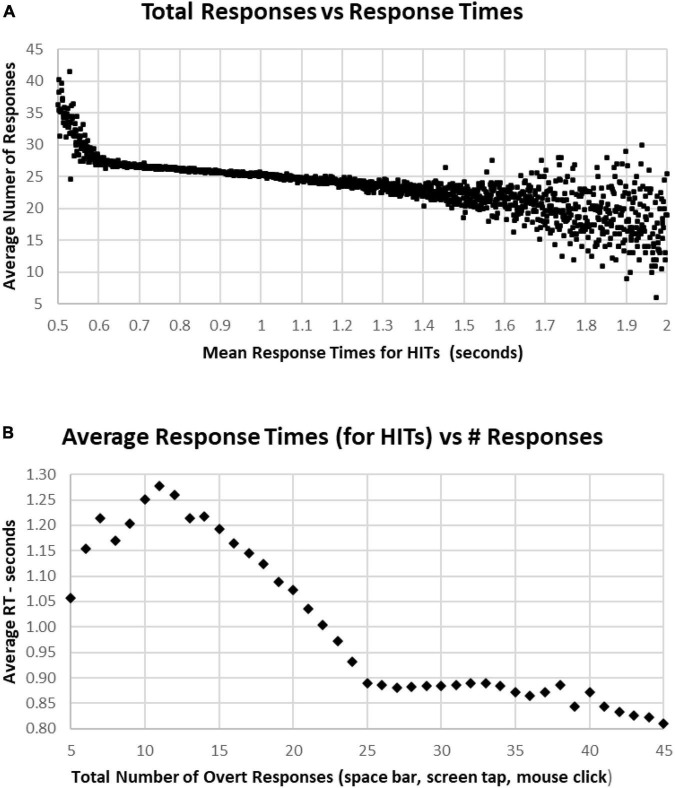
**(A)** Average number of responses for each HIT RT, showing that there is an increase in the average under 0.600 s, while there is an increase of variability for RTs longer than 1.5 s. The discordance between number of responses and decreased RT below 0.6 s is associated with a strategy to respond more quickly, but with less discrimination. **(B)** Same data, averaging RTs by number of responses. Note sharp change of slope below the 25-response mark, with progressive slowing until 10 or fewer responses. But there is a stable level of RTs with an increased number of responses, suggesting that by increasing the number of false alarms, that this component of accuracy was sacrificed for speed.

Percentile = 1-EXP(–263.94*EXP(–6.682*RT))

for RTs for HITs between 0.6 and 1.6 s, with 0.5% of the data above and the same number below these limits. Only 0.2% of the responses during these tests were above 1.8 s, and those RTs were chaotic (Figure 9A). Backward calculating this curve to create a reverse exponential distribution (RevEx) and superimposing this curve on the distribution of RTs for HITs shows essentially a perfect fit, considering some statistical noise and a deterioration of performance for RTs greater than 1.6 s (Figure 8B). Note that the Rev-Ex model requires two parameters while the ex-Gaussian requires three parameters.

### Relationship between correctness of responses and response time

In general, faster RTs were associated with individual tests having above the optimal number of responses (25), with a sharp increase of number of responses for those in the fastest 2% (below 2 standard deviations, 0.64 s), and a progressive decrease of responses with higher RTs, down to an average of 22 responses at 1.5 s. There was a scattering of RTs among the slowest 2% (above 2 standard deviations, 1.4 s) (Figure 9A). Of note, apropos to the variation of performance/strategy for those having more or less than 25 responses, those tests with more than 25 responses were associated with progressively faster RTs from 0.9 s for 25 responses and 0.8 s for 45 responses (Figure 9B). Alternatively, fewer than 25 responses were associated with a progressive slowing of responses to 1.278 s for 11 responses. For the 969 subjects with 5–10 responses, RTs decreased progressively to 1.05 s for only five responses (average of 127 tests).

**FIGURE 10:**
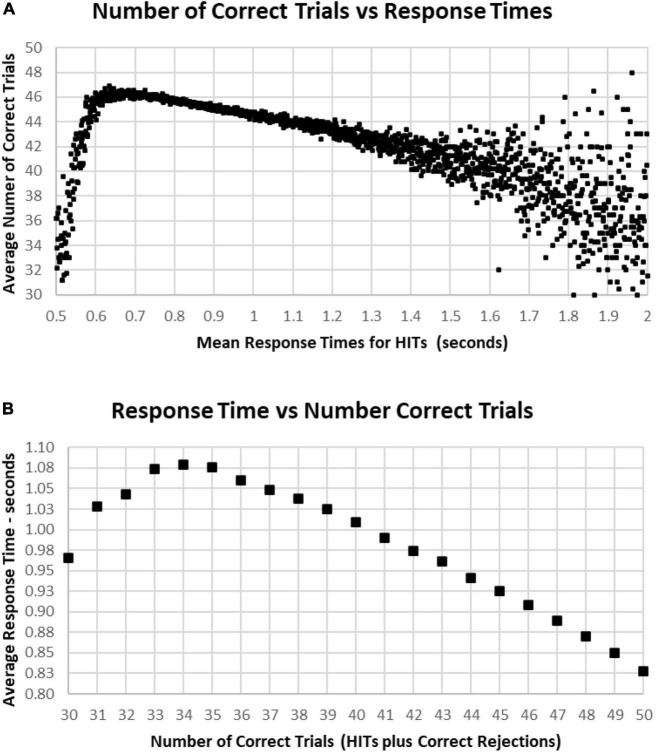
**(A)** Average number of correct responses for each RT. The most correct responses occurred at an RT of about 0.650 s with about 46 correct (90%). The average number correct fell sharply with faster RTs and more slowly with slower RTs, until an increased variability is seen, largely due to the smaller number of subjects with RTs slower than 1.4 s. **(B)** Again, same data averaging RT by number of correct responses. Note that 100% correct (50 correct responses) is associated with an RT of 0.828 s. With a decreased number of correct responses, there is a progressive slowing of RT until an RT of 1.079 s at 34 correct (68%), but lower numbers of correct responses again show the discordance of RTs and performance with poorer levels of performance.

The most correct performances were associated with RTs of 0.6–1.0 s, with the average number of correct trials for this range being 44–46 (HITs plus Correct Rejections, trials with 2–6 errors) (Figure 10A). Progressively faster RTs from 0.6 to 0.5 s were associated with a rapid deterioration of correct performance to chance, while RTs from 1.0 to 1.5 s were associated with a more gradual deterioration of correct responses to 40 (10 errors). Less than 1% of participants had RTs over 1.5 s, and these subjects had a broad range of correct responses, 30–48 (2–20 errors) (Figure 10A). Importantly, those subjects with perfect scores (0 errors) averaged 0.84 s, with increasing errors associated with progressively slower RTs for HITs, so that the average RT for those subjects having 35 correct trials (with 15 errors) was 1.07 s (Figure 10B). Below this level of performance, those having 30–35 correct trials (with 15–20 errors) had slightly faster RTs, which was associated with the increased variability of performance in this lower 3% of the group.

**FIGURE 11:**
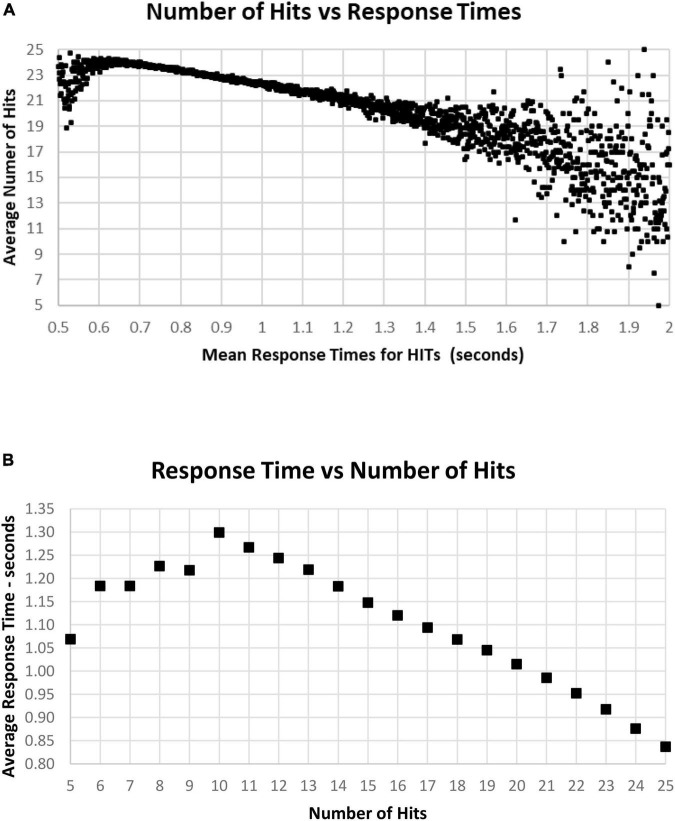
**(A)** Average number of HITs for each RT, showing the HIT component of correct responses. Note that the most rapid average RT is about 0.650 with an average of 24 HITs. There is a relatively small number of faster responses, most with over 21 HITs. Beyond 1.4 s, there is again a smaller number of tests, and the wide distribution of responses reflects that smaller number. **(B)** Same data as **(A)**, showing the optimal number of HITs, 25, is associated with an average RT of 0.837 s, with a progressive slowing associated with fewer HITs to an RT of 1.299 s at 10 HITs. With a smaller number of HITs, there is a faster RT, reflecting a lower level of discrimination.

As noted above, there is a considerable division in behavior for HITs and Correct Rejections. As the data for correct performance is a sum of HITs and Correction Rejections, there is a substantial question of how these measures relate to RT. When RT to HITs was compared only to the number of HITs, there was a closer relationship than with number of responses or total correct trials (Figure 11A). The average number of HITs was consistently at least 20 out of 25 for RTs between 0.5 and 1.4 s. The relationship between HITs and RTs showed that the optimal number of HITs, 25, was associated with a RT of 0.837 s, with a smooth slowing, decreasing HITs to 10 at a response time of 1.3 s (Figure 11B). For the 1,083 tests with only 5–9 HITs, RT was then progressively faster to 1.069 s.

**FIGURE 12:**
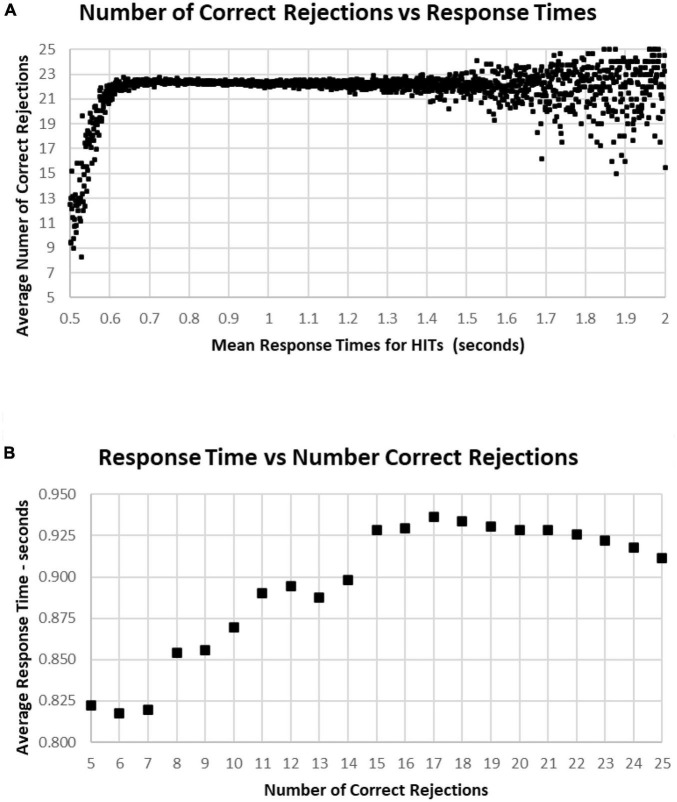
**(A)** Average number of Correct Rejections for each RT, showing the Correct Rejection of component of correct responses. Note that that over an RT of 0.650 s, there is essentially no relationship of Correct Rejections and RT, with about 22 Correct Rejections occurring on the average. By contrast, for faster RTs, there is a steep speed/accuracy trade-off between speed and accuracy of Correct Rejections. Again, the small number of tests above 1.4 s show the dispersion with fewer tests, but there is no indication of a different slope. **(B)** Again, same data as **(A)**, showing an RT of 0.911 s for 25 Correct Rejections and a slight increase of RT down to 15 Correct Rejection, but there are faster RTs with lower numbers of Correct Rejections, which reflects the alteration of strategy (less inhibition) associated with this aspect of poor performance on the MemTrax test.

**FIGURE 13:**
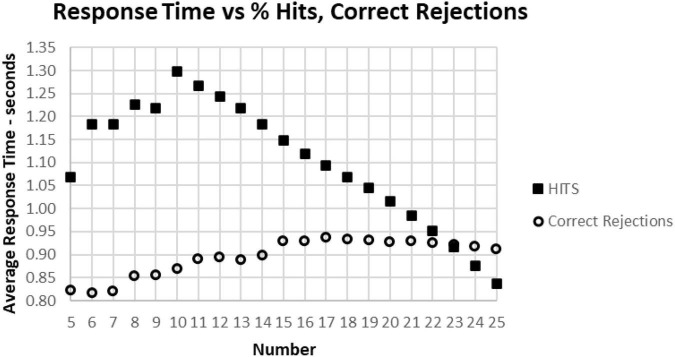
The average correct response times broken down into True Positive and True Negative groups. Note that the number of True Positive responses has a clear linear relationship with the average response time. The True Negative choices have very little effect when there are more than 15 (less than 10 false positive responses), but they have a negative relationship with RT below 15, suggesting that the increased number of false responses is related to making faster incorrect responses.

A major factor associated with RT was response bias, the tendency to make fewer than or more than 25 responses (the ideal number being 25). This tendency is most clearly seen when examining the Correct Rejections. The RT had relatively little relationship with Correct Rejection count (Figure 12A). For the 95% of tests with RTs between 0.64 and 1.4 s, the number of Correct Rejections was very stably close to 22. However, for RTs less than 0.640 s, there was a clear, sharp drop in the number of Correct Rejections. Above 1.4 s, there was again a scattered pattern of Correct Rejections with no consistent relationship with RT. When the averages of RT were compared for numbers of Correct Rejections, there was only a slight slowing from the optimal number of 25 at 0.910 s to around 930 ms for only 15 correct, showing the minimal relationship between RT and Correct Rejections for tests with better performance (Figure 12B). However, for tests with only 5–14 Correct Rejections, there was the progressive shortening of RTs with fewer Correct Rejections again seen. Clearly, the pattern of the relationship between Correct Rejections and RT was very different than the one between HITs and RT (and shown on the same axis to highlight the difference, Figure 13).

Figures 9–12 show the complex relationship between correctness of responses and response tendency to RT. To determine the Pearson correlation between correct responses and RT, the major outliers were removed. Tests with fewer than 10 HITs (1,083), fewer than 15 Correct Rejections (5,400), RT less than 600 ms (1,538), or more than 1.4 s (5,856), and trials with less than 35 correct responses (1,277) were eliminated (total removed = 15,565 = 5%), leaving 266,584 tests. This removal (had minimal effect on the correlations) produced a correlation between RTs and Correct Responses: *R*^2^ = 0.081, while the correlation between RTs and HITs: *R*^2^ = 0.14, was significantly higher. When averaging across RTs for each number of correct responses (35–50) or HITs, 10–25, there was a clear linear progression seen, which is like the curves of Figures 10B, 11B, with a linear regression explaining essentially all of the variance for 35–50 correct responses and 10–25 HITs, respectively. The clear emergence of this high explanation of the variance indicates that the MemTrax test is measuring important neurophysiological phenomena in visual information processing, but there was a substantial amount of noise when assessing individual subject performances.

## Discussion

The present study demonstrated that the MemTrax CRT – an inexpensive and scalable platform – can be efficiently used to obtain a large amount of reliable behavioral data describing learning, memory, and cognition in populations, with important implications for cognitive performance metrics for individuals. By selecting performances meeting non-random criteria and appropriate response characteristics, population distributions of data could be analyzed and compared. The MemTrax CRT data contained measures of correctness, Total Number Correct, HITs, Correct Rejections, False Alarms, and RTs. The first principal finding was that HITs and Correct Rejections did not correlate with each other, meaning that Signal Detection Theory analysis would not apply, and HITs and Correct Rejection accuracy had very different implications for explaining a subject’s performance. The second principal finding was that the average RTs corresponded more closely with HITs than overall correctness or Correct Rejections. The third principal finding was that the RT distribution followed a reverse-exponential (RevEx) model requiring only two parameters.

Previous studies have shown effects of age and education on these MemTrax metrics (Ashford et al., 2011, Ashford et al., 2019b). Further, two comparisons with the popular cognitive screening test, the Montreal Cognitive Assessment (MoCA), have shown MemTrax to perform at least as well for distinguishing cognitive impairment from normal function using a more efficient system (Liu et al., 2021), and MemTrax RT significantly correlated with six of the eight domains measured by the MoCA, visuospatial, naming, attention, language, and abstraction (van der Hoek et al., 2019). The MemTrax test has also been evaluated using machine learning showing relationships with other health measures (Bergeron et al., 2019). This study extends the findings of these and other prior studies using a very large population. The large population, with data selected from 344,165 presumed-unique, anonymous users, provides performances of nearly every possible variety, and reflects the behavioral diversity of the online population, which is becoming more and more representative of the whole population. And the large number of users and repeat uses reflects the degree to which this test is highly engaging. These analyses showed that both HIT responses, Correct Rejections, and RTs for those HITs to stimuli repeated can be measured and used in large projects.

### Response accuracy

Of specific interest were the analyses of correct responses (HITs and Correct Rejections) and incorrect responses (False Alarms and Misses) from 282,140 users who performed this CRT paradigm within acceptable levels. The tests selected for analysis were from first-time users in which at least 30 out of 50 trials were correct, which indicated non-random performance. Further, tests were selected from which mean RTs for HITs on the correct trials were between 0.5 and 2.0 s, indicating reasonable efforts by the users. The requirement for at least 30 correct trials assured that there would be at least five HITs or five Correct Rejections on a test.

An important finding was the lack of a correspondence between HITs and Correct Rejections. Obviously, overall correct performance is an addition of HITs and Correct Rejections, so each will correlate with total correct. But when looking across the whole population, these two metrics had no correlation with each other (Figure 5A). Accordingly, these two independent metrics appear to represent distinct phenomena, reflecting the information processing challenges of the test and the strategy for optimizing the balance between accuracy and speed. The performance of a HIT requires recognition of a prior image, successful access of STM, and the decision to respond affirmatively. However, if there is uncertainty about the recognition or confusion with a similar image which is new, a blurring of episodic memory, a response will be a False Alarm. Because of the constant variation of stimuli and categories, HITs will reflect the level of certainty about a repeated image. Alternatively, a False Alarm will reflect a bias to respond with a lower level of certainly. Thus, the number of correct responses does not reflect a “signal detection,” a degree of differentiation and a response bias. Instead, HITs reflect a recognition threshold, and False Alarms represent an error threshold. The response strategy can then reflect an effort to respond only when there is a high level of certainly or to respond to avoid missing any targets.

With respect to interpreting MemTrax performance, the occurrence of Miss errors and False Alarm errors provides information which must be managed when interpreting the MemTrax performance metrics. Understanding precise relationships between correct and incorrect responses within the MemTrax test provides information to improve its applicability to screening and assessment of learning, memory, and cognitive functions in clinical settings.

### Response time distribution

Of particular interest was the skewed distribution of RTs for HITs. Analysis of the MemTrax data, at least for this population, showed a distribution with a skewed slope for averaged RTs for HITs, which was very steep for rapid RTs and particularly long for slower RTs. The analysis of the data from the present study showed an exponential function, the reverse of a survival curve, RevEx, could fully explain the variance of the RT distribution skew. This exponential function can be interpreted as a requirement for doubling the processing power for every 100 ms of decrease in RT. This pattern suggests that the nervous system must double the resources expended to analyze and respond to the complex information in the presented stimulus for each 100 ms unit of time faster, or conversely, halving the neuronal resources would slow the RT by 100 ms. This exponential increase of resources required for shortening RT explains why it is essentially impossible to respond faster than 0.5 s and maintain correct responses.

A variety of theoretical explanations have been invoked to explain this skewed distribution for RTs across many paradigms (Ratcliff et al., 2016; Moret-Tatay et al., 2021). However, the RevEx model provides a different and direct reflection of the massive, reciprocal processing capability of the brain, without reliance on concepts of a series of processing stages. This insight is consistent with neurophysiological analyses of neuron responses showing simultaneous neuronal processing across broad reciprocally connected cortical and brainstem regions (Ashford and Fuster, 1985; Coburn et al., 1990; Ashford et al., 1998a) and cannot be deduced from IPMs proposing a series of processing steps. This perspective of RTs may have important applications for identifying contributors to normal and abnormal processing. Further, the slowing of RT with neurodegeneration can be linked to the loss of neural network resources, as occurs in AD (Gordon and Carson, 1990; Ratcliff et al., 2021).

This skewed pattern of these RTs has a mathematical relationship to the survival curve of essentially all living things discovered by Benjamin Gompertz in 1825, referred to as the Gompertz Law of Aging (Hirsch, 1997; Gavrilov and Gavrilova, 2001; Ashford, 2004; Raber et al., 2004; Ashford et al., 2005), just in reverse. The survival curve of all living beings is related to an exponentially increasing rate of mortality with age. This “fact of life” has been interpreted as describing an exponentially increasing rate of failures across massively redundant systems; but by contrast, the Weibull curve applies to mechanical systems, not living systems (Gavrilov and Gavrilova, 2001). The exponential increase of failures occurs in a progressively more rapidly dwindling population that leads to the appearance of a sharp rate of population decline in extreme age. These MemTrax data showed that the skewed RT distribution curve is most efficiently explained by an exponential increase of demand for information processing resources to shorten RT, a reverse-exponential (RevEx) function. The RevEx interpretation accurately describes how reducing resources in a working, learning, memory, and cognitive neurophysiological system, or information processing failures, slows RT, while implicit or explicit recruiting of additional resources to analyze and respond to the incoming information leads to a more rapid RT (Kahneman, 1973). Critically, exponentially increasing recruitment of resources initially shortens RT but finally exhausts the neural resources available for processing, so accurate RTs are nearly impossible to achieve for less than about 0.6 sec in the MemTrax CRT. The RevEx model provides a skewed RT distribution with two easily derived parameters. This curve can be used as a reference continuum describing the scale of severity against which individual responses can be compared and is likely applicable in all such information processing studies examining RT.

### Relationship between performance correctness and response time

Examining various response correctness patterns in relationship to the distribution of RTs, suggests that part of the processing reflected variations in error-inducing strategies. The early part of the RT distribution appears to reflect a bias to respond (more False Alarms) that reduces time to process information and leads to more errors thus shortening the RTs for HITs. As the strategy becomes less about distinguishing between new and repeated images and more about rapid response, showing a speed/accuracy trade-off in this narrow range, with the average number of Correct Rejections dropping to 9. However, with slower RTs there is a clear relationship to decrease of HITs, reflecting the failure to either encode or recognize repeated images and taking exponentially longer to process the visual information. This analysis is particularly relevant for identifying progressive loss of synaptic connections, as seen in aging and AD (Gordon and Carson, 1990; Ratcliff et al., 2021), which are accompanied by a retrogenesis of the neurons (Ashford et al., 1998b; Ashford and Bayley, 2013) and changes in control of executive function (Yesavage et al., 2011). These effects likely alter neuroplasticity and the efficiency of the information processing sequence and the resources available for encoding and recognizing item information over the duration of the task (Ashford and Jarvik, 1985; Coleman and Yao, 2003; Ashford, 2019). Accordingly, each 50% loss of neural processing capacity would slow RT by about 100 ms.

The overall balance of HITS and Correct Rejections and the interaction with RT play critical roles in strategy and analytic ability. The False Alarms metrics were not associated with RT, except at the shortest 1% of RTs, where increasing False Alarms were associated with a speed/accuracy trade-off. At the fastest RTs, there is a likelihood that the participant was utilizing a strategy that made decisions so rapidly that adequate analysis of the image was not occurring. However, at more usual and slower RTs, False Alarms, which are unrelated to RT, likely represent the failure of response inhibition, responding to a new stimulus falsely processing it as a repeated stimulus. Consequently, False Alarms without a speed/accuracy trade-off likely represent failure of frontal lobe inhibitory function, as has been seen clinically in patients with diagnoses of fronto-temporal dementia (JWA, clinical observation). Alternatively, an increased number of Misses was related to a progressive slowing of RT, which likely represents a slowing of the occipital-temporal-hippocampal visual system to process information, with increasing difficulty recognizing repeated visual information and generating a recognition response. The latter condition explains the impairment of patients with mild cognitive impairment, such as AD (JWA, clinical observation), and other conditions affecting the temporal lobe.

An additional finding was the low correlation between HITs and RTs across the population, explaining 14% of the variance (the opposite of a speed/accuracy trade-off – more HITs was related to faster RTs); but when RTs were averaged for specific HIT-rates, there was a nearly perfect relationship between decreasing HITs and increasing RT, with a loss of about 30 ms per additional miss from 25 HITs to 10 HITs. The relationship between RT and average HIT-rates explained nearly 100% of the variance of the averages, suggesting a significant phenomenon. Accordingly, about 85% of the variance in the relationship between HITs and RTs was related to variables aligned with the state of the individuals at the time of testing. In principle, this individual variance could be reduced by repetitions of the MemTrax test, which can be done essentially without limit, and frequent administrations of the MemTrax test over time. Monitoring the relationship between HITs and RTs could accurately assess changes in the function of patients, related either to disease progression or treatment benefits.

The present analysis indicates that HITs, the instances in which a repeated stimulus is recognized, has a relationship with the response speed – not the tendency to under- or over-respond. Thus, False Alarms alter the relationship between RTs and HITs. The relationship between total number of responses and more subjects with faster RTs in Figure 10A, descending from 1.5 to 0.6 s, an improvement of speed with more responses, is not due to a speed/accuracy trade-off; but this relationship is complex and related to a portion of participants over-responding to new stimuli. However, the increased correlation after accounting for the over-response tendency indicates that there is a significant positive relationship between RTs and correct responses, not a speed/accuracy trade-off.

The time to respond (RT) to stimuli has been used as a dependent variable to study effects of non-clinical and clinical phenomena on learning, memory, and cognitive functions. Complex picture recognition has been particularly useful for studying medial temporal lobe function (Koen et al., 2017) and neural responses in the human hippocampus are related to episodic memory (Suzuki et al., 2011; Wixted et al., 2018). A cross-species study using this strategy showed that pigeons and monkeys were able to recall complex pictures moderately well, but that humans remembered pictures so well that it was necessary to utilize kaleidoscope images to test the limits of human memory and recall and recognition (Wright et al., 1985).

Information processing models provide a neurological and psychological structure to conceptualize distributions of behavioral indices which occur during a subject’s performance of a task (Broadbent, 1965). A stimulus presented on a trial interacts with and engages numerous processes in the brain that sense and provide a rapid, modality-dependent analysis of the physical parameters of that stimulus. This initial sensory analysis engages attention and temporarily represents this stimulus in limited capacity STM, for determination of whether that information has been presented previously. If the stimulus is analyzed as being novel, then the information about that stimulus is transduced, integrated, associated, and consolidated with other items previously consolidated into the massive capacity memory storage system, LTM (Atkinson and Shiffrin, 1971) for later use. And it is the initial presentation in which these processes are occurring, with activation of the hippocampus, not during the later recognition (Suzuki et al., 2011). Occurrences in STM can interact with processes directed by operations in WM, a space where these events can be manipulated (Baddeley et al., 2019).

Instructions provided to the individual before the task began directed the information processing operations to execute cognitive mechanisms addressing those events in STM that satisfied task demands on a trial. Occurrences in STM also engage processes that consolidate this information and associate and integrate it with information previously stored in LTM. LTM consists of an associative neural network that inter-relates all items in LTM. This information in LTM is continuously recalled and integrated with that in STM to support perceptual stability.

Long-term memory is divided into declarative and non-declarative subcomponents. Declarative, also referred to as explicit memory, refers to information stored in LTM which can be intentionally recalled into STM, where that memory trace can be retained for seconds or minutes, but STM capacity is severely limited by exposure to additional information. A person can intentionally direct those operations in WM to execute processes that maintain the quality and distinctiveness of this occurrence in STM, but only for a limited duration. Information must be transferred into LTM systems for recognition or recall beyond the STM capacity.

Behavioral patterns during these tests may separate healthy individuals from those with memory declines produced by mild cognitive impairment (Stark et al., 2013) and AD (Ashford et al., 1989; Ashford, 2008). This memory loss occurs 5–10 years before dementia diagnosis associated with AD (Tierney et al., 2005). AD impairs recognition of complex pictures after even a brief delay. This rapid loss of perceived information may reflect effects that AD has on neurological structures subserving memory, specifically, impairment of neuroplasticity (Ashford and Jarvik, 1985; Ashford, 2019). Distinguishing different modes of processing may produce data that improves clinical classification of various aspects of dementia and AD.

Response time during performance of a CRT has been used to study effects of AD on learning, memory, and cognition. Simple reaction time to stimuli is relatively preserved in AD patients with mild impairment, while choice reaction time to difference between stimuli is adversely affected (Pirozzolo et al., 1981). AD patients show substantial slowing of RT in cognitive tasks (Mahurin and Pirozzolo, 1993) requiring this maintenance of information in STM during testing. Extensive study of RTs to different types of stimuli in the elderly has shown a general proportional linear increase in RT toward those stimuli, with a disproportionate deterioration of those RTs related to memory function (Poon and Fozard, 1980; Bowles and Poon, 1982; Hines et al., 1982; Myerson et al., 1990; Yesavage et al., 1999; Ishihara et al., 2002). Consequently, these MemTrax modifications of the CRT paradigm could be of considerable utility for addressing within- and between-trial and test-retest variation that enables tracking effects that pathology has on learning, memory, and cognitive function. Evaluating such functions with computer testing can add considerable precision for early detection of clinical phenomena when the impairment is still mild along the continuum to later and more severe states.

Similar tests administered in-person, the Continuous Visual Memory Test (Larrabee et al., 1992) and the Continuous Recognition Memory test (Fuchs et al., 1999), have already been shown to have construct validity. Of note, the Continuous Recognition Memory test shows the same dissociation of HITs and Correct Rejections shown for these MemTrax data. Substantial enhancement of the applicability of MemTrax can be achieved by analyzing the several MemTrax variables and establishing their precise relationships with respect to externally obtained data and integration with other systems such as is being conducted by the Brain Health Registry (Mackin et al., 2018; Weiner et al., 2018; Cholerton et al., 2019) and factor analysis with respect to the presence and severity of organic brain dysfunction and dementia (Larrabee, 2015; Ratcliff et al., 2021). This study has shown the potential utility of the MemTrax on-line CRT for gathering information about learning, memory, and cognitive status of users. Preliminary analyses of data from the French company HAPPYneuron and the Brain Health Registry have shown similar results, indicating the generalizability of these data.

## Limitations

The MemTrax data analysis presented here is from anonymous users in a convenience sample, and therefore cannot be considered validation for any specific purpose. However, the data do clearly show a distribution of responses consistent with prior studies which have included verified data. Therefore, the data and analysis provided here is likely an acceptable approximation of what would be expected in a representative sample of the general population. Table 1 provides the tabulated data and percentiles from this anonymous group and provides precision measures to support the published studies showing the validity of MemTrax.

**TABLE 1:** Tabulated data for 282,140 on-line MemTrax users, with percentiles (%iles).

%C	#C	%ile	# HITs	%ile	#CRs	%ile	RT	%ile
60	30	0.30%	9	0.30%	9	0.30%	0.5	100.00%
62	31	0.50%	10	0.40%	10	0.50%	0.6	99.20%
64	32	1.00%	11	0.50%	11	0.70%	0.7	91.40%
66	33	1.40%	12	0.70%	12	0.90%	0.8	71.60%
68	34	1.80%	13	0.90%	13	1.10%	0.9	47.50%
70	35	2.30%	14	1.20%	14	1.50%	1	28.20%
72	36	2.90%	15	1.70%	15	1.90%	1.1	15.60%
74	37	3.70%	16	2.30%	16	2.60%	1.2	8.30%
76	38	4.90%	17	3.30%	17	3.50%	1.3	4.40%
78	39	6.40%	18	4.80%	18	5.10%	1.4	2.30%
80	40	8.60%	19	6.90%	19	7.70%	1.5	1.20%
82	41	11.50%	20	10.10%	20	11.90%	1.6	0.60%
84	42	15.50%	21	15.10%	21	18.60%	1.7	0.30%
86	43	20.90%	22	22.80%	22	28.60%	1.8	0.20%
88	44	28.00%	23	34.50%	23	43.10%	1.9	0.10%
90	45	37.10%	24	52.10%	24	61.70%	2	0.00%
92	46	47.90%	25	76.60%	25	82.80%		
94	47	60.10%						
96	48	72.90%						
98	49	85.10%						
100	50	94.90%						

Further analyses are needed to determine how numerous factors, including age, sex, education, apolipoprotein-E and other relevant genetic factors, and clinical conditions relate specifically to the MemTrax parameters across numerous populations (Bergeron et al., 2019; Zhou and Ashford, 2019). MemTrax analysis with “machine learning” can further and more definitively classify cognitive function (Bergeron et al., 2020).

MemTrax data sets particularly include all the RTs for each subject’s response and analysis of intraindividual variability in RT may also represent an important indicator of performance (Tse et al., 2010; Kennedy et al., 2013; Cousineau et al., 2016). While individual RTs can be easily and simply analyzed for each subject and then related to the RevEx model, additional analyses are needed to determine an individual’s level of cognitive function or dysfunction more precisely.

In the MemTrax CRT, there is a variable lag between initial and repeated presentations, which can affect memory encoding (Hockley, 1982; Ashford et al., 2011). The degree of that effect, including the number of intervening items between initial and repeat presentations as well as the position of the repeat in the 50-item continuum, was not analyzed here, but this metric can be assessed as was previously shown (Ashford et al., 2011). Further, there are five items, one from each of the five categories, repeated a second time, and the degree of strengthening of the encoding of the doubly repeated items can be assessed (Hintzman, 2016). Advancing the analytic development of this CRT paradigm may lead to even more powerful assessments.

Given the global accessibility to the internet, there is essentially no verifiable information about the subjects with this isolated web-based testing that can be used as additional independent variables. For example, when asked to provide year of birth, of 344,165 presumed unique individuals who completed a test only 26,834 provided year of birth and even this information could not be independently verified in this study, though this factor was available from other studies of MemTrax and did show an age effect (Ashford et al., 2019b). Accordingly, select demographic and clinical data must be obtained through other means to further examine epidemiological effects on data during CRTs and establish clinical utility. Data from the Brain Healthy Registry, which provides MemTrax as one of its assessment tools (Cholerton et al., 2019; Nosheny et al., 2020), has demographic and cognitive function information for comparison, and such analyses are planned. As noted above, MemTrax variables percent correct on RT significantly correlated with six of eight MoCA domains (van der Hoek et al., 2019), and adding HITs and correct rejections and with different images and different performance instruction, numerous other cognitive and cortical domains could be assessed with this platform.

The data presented here cannot be construed as representing a properly sampled population. With no clinical information, there was no clinical validation; thus, there was only a suggestion of what likely clinical indices would be. For example, 2.2% of the population (consider 2 standard-deviations) had less than 70% correct, 15 HITs, 15 Correct Rejections and RT slower than 1.4 s. For a cut-point for less impairment, 6.7% of the population (consider 1.5 standard-deviations) had less than 78% correct, 18 HITs, 18 Correct Rejections, and RTs slower than 1.23 s. For a cut-point for less impairment, 15.7% of the population (consider 1 standard-deviation) had less than 84% correct, 20 HITs, 20 Correct Rejections, and RTs slower than 1.1 s. Data from Table 1 could be used to estimate performance levels below 1 or 1.5 deviations below the mean. Tests with fewer than 15 Correct Rejections (more than 10 False Alarms) can be considered invalid and may represent frontal-lobe dysfunction.

## Conclusion

Prior studies on mild cognitive impairment (Koppara et al., 2015; Ratcliff et al., 2021) and AD (Gordon and Carson, 1990; Schumacher et al., 2019) have already shown the potential of the CRT approach for assessing disorders of learning, memory, and cognition. However, the analysis of the MemTrax data provided a different perspective on cognitive function than has been based on SDT methods alone and provided a novel perspective for understanding cognition and memory, revealing levels of complexity beyond the traditional paradigms. Moreover, MemTrax has been shown to provide at least as much information as the Montreal Cognitive Assessment (MoCA) (van der Hoek et al., 2019; Liu et al., 2021). The precision provided by MemTrax also suggests that MemTrax could improve the specification of the severity of cognitive impairment in early phases of AD (Ashford et al., 1995, Ashford et al., 2019a; Ashford and Schmitt, 2001; Ashford, 2008), as well as the pace of change over time with repeat testing. By assessing performance metrics and RT, MemTrax also has the capability to screen for many varieties of cognitive impairment and would be an ideal tool for use in the elderly US population for the Medicare Annual Wellness Visit (Ashford et al., 2022). However, more testing in clinical populations is needed to implement on-line testing for broad clinical applicability and widespread screening.

## Data availability statement

The raw data supporting the conclusions of this article will be made available by the authors, without undue reservation.

## Ethics statement

The studies involving human participants were reviewed and approved by Internal Review Board (IRB), Stanford University. Written informed consent from the participants’ legal guardian/next of kin was not required to participate in this study in accordance with the national legislation and the institutional requirements.

## Author contributions

JA did the analyses, produced the figures, and wrote the first draft of the manuscript. JC worked extensively on developing the presented concepts. SA worked with JC on the concepts. MB provided guidance, writing, and extensive editing. PB provided expert consultation on the psychological test theories, including writing and editing. CA implemented the CRT at https://memtrax.com, managed the website, and recruited all the users who took the test. All authors contributed to the article and approved the submitted version.
